# Sex differences in the human peripheral blood transcriptome

**DOI:** 10.1186/1471-2164-15-33

**Published:** 2014-01-17

**Authors:** Rick Jansen, Sandra Batista, Andrew I Brooks, Jay A Tischfield, Gonneke Willemsen, Gerard van Grootheest, Jouke-Jan Hottenga, Yuri Milaneschi, Hamdi Mbarek, Vered Madar, Wouter Peyrot, Jacqueline M Vink, Cor L Verweij, Eco JC de Geus, Johannes H Smit, Fred A Wright, Patrick F Sullivan, Dorret I Boomsma, Brenda WJH Penninx

**Affiliations:** 1 Department of Psychiatry, VU University Medical Center, Neuroscience Campus Amsterdam, Amsterdam, The Netherlands; 2 Department of Biological Psychology, VU University Amsterdam, Neuroscience Campus Amsterdam, Amsterdam, The Netherlands; 3 Department of Biostatistics, University of North Carolina, North Carolina, USA; 4 Department of Genetics and the Human Genetics Institute, Rutgers University Cell and DNA Repository, Rutgers University, New Jersey, USA; 5 Departments of Genetics and Psychiatry, University of North Carolina, North Carolina, USA; 6 Department of Pathology, VU University Medical Center, Amsterdam, The Netherlands

## Abstract

**Background:**

Genomes of men and women differ in only a limited number of genes located on the sex chromosomes, whereas the transcriptome is far more sex-specific. Identification of sex-biased gene expression will contribute to understanding the molecular basis of sex-differences in complex traits and common diseases.

**Results:**

Sex differences in the human peripheral blood transcriptome were characterized using microarrays in 5,241 subjects, accounting for menopause status and hormonal contraceptive use. Sex-specific expression was observed for 582 autosomal genes, of which 57.7% was upregulated in women (female-biased genes). Female-biased genes were enriched for several immune system GO categories, genes linked to rheumatoid arthritis (16%) and genes regulated by estrogen (18%). Male-biased genes were enriched for genes linked to renal cancer (9%). Sex-differences in gene expression were smaller in postmenopausal women, larger in women using hormonal contraceptives and not caused by sex-specific eQTLs, confirming the role of estrogen in regulating sex-biased genes.

**Conclusions:**

This study indicates that sex-bias in gene expression is extensive and may underlie sex-differences in the prevalence of common diseases.

## Background

Sexual dimorphism extends into marked cellular, metabolic, physiological and anatomical differences and leads to sex differences in disease prevalence, expression and severity of, for example, cardiovascular [1], and autoimmune [2] diseases, personality [3] and psychiatric disorders [4]. Sex inequalities are an increasingly recognized challenge in both basic research and clinical medicine [5], and understanding the molecular mechanisms behind sex differences may lead to new insights into sex-specific pathophysiology and treatment opportunities [6].

Sex differences at the DNA sequence level are restricted to the sex chromosomes. On the X-chromosome, most genes are equally expressed across sex due to X-inactivation in women [7]. The few unshared genes located on the Y chromosome are exclusively expressed in the testes, or are housekeeping genes with X-chromosome homologues that escape X-inactivation [8]. However, genome regulation seems highly sex-specific at secondary epigenetic levels such as DNA methylation [9], DNase hypersensitivity [10], chromatin structure [11] and gene expression [12,13]. Thus, a characterization of sex differences in genome regulation by gene expression will contribute to the understanding of the molecular basis of sexual dimorphism.

Animal studies have shown that sex-biased gene expression is highly tissue dependent [14,15] and the evolution rates of sex-biased genes are higher than average [12,16]. Two recent studies in mice reported sex differences in gene expression networks of correlated transcripts [17,18]. Surprisingly few studies aimed at identifying and investigating sex-biased genes in humans, and only in small sample sizes (*N* < 250 [19-22]). Nonetheless, consistent evidence was obtained for sex-specific gene expression.

Sex-differences in gene expression will depend on the hormonal status of the group considered. For instance, during menopause, much of the female-specific hormone production ceases, with downstream effects on gene expression in adipose tissue [23], monocytes [24], and bone [25]. In women using hormonal contraceptives, containing the hormones estrogen and progesterone, additional differences in gene expression may be evident as well.

For many genes, expression levels are influenced by DNA polymorphisms (eQTLs). Although the sexes do not differ at the autosomal DNA sequence level, sex differences in gene expression may be caused by sex-specific eQTLs [26] (i.e. some SNPs may influence gene expression in one sex, but not in the other).

Here we used microarrays to identify genome-wide sex-biased gene expression in the human peripheral blood transcriptome in a large sample (*N* = 5241 subjects) from the Netherlands. The sample size was sufficiently large to account for menopause status and hormonal contraceptive use. The identified sex-biased genes were characterized in terms of enrichment for functional gene ontology (GO) and disease categories, distribution across the autosomes and sex chromosomes, tissue specificity, evolution rates, participation in major gene expression networks and the extent to which sex differences in gene expression were caused by sex-specific eQTLs.

## Results

### Sample description

The sample consisted of 5,241 individuals from the Netherlands Study of Depression and Anxiety (NESDA) and Netherlands Twin Register (NTR) cohorts (Table 1; [27]). Of the women, 22% were postmenopausal and 31% used hormonal contraceptives. For all participants, genome-wide gene expression in peripheral blood was assessed using microarrays with 47,122 probe sets targeting 19,250 genes. For each probe set, mixed models including demographic, and several technical covariates were used to test for sex effects (see Methods).

**Table 1:** Demographic summary of sample

	**Female**	**Male**
Total # subjects (after QC)	3427	1814
group (NTR/NESDA)	2079/1348	1147/667
age (mean/sd)	38.5/12.7	38.9/13.7
bmi (mean/sd)	25.6/4.1	24.5/4.6
smoking status (percentage of smokers)	27.00%	32.00%
red blood cell count	8.3/0.6	9.4/0.6
Menopause status females (pre/post)	2687/740	
Contraceptive pill use (yes/no)	1093/2334	

The sample consisted of 5241 subjects (after QC) from the Dutch NESDA and NTR cohorts.

### Sex effects on gene expression

**Figure 1:**
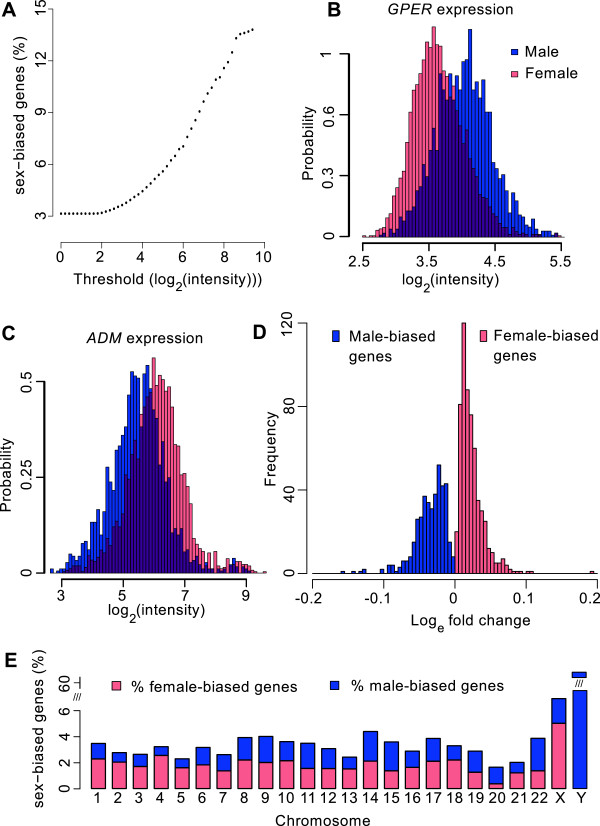
**Characterization of female- and male-biased genes.** For each of the 47,122 transcripts the sex effect was determined using a mixed model, resulting in 3.1% sex-biased genes. **A)** Transcripts were selected based on a threshold for mean expression, the percentage of sex-biased genes increases with the threshold that is used: in genes that are highly expressed there are more (up to 13%) sex-biased genes than in genes that have low expression. Nonetheless, also large male/female fold changes were observed in genes with low **(B)** and moderate **(C)** expression. **D)** For each transcript fold changes were computed; on the autosomes 57.7% of the sex-biased genes was female-biased, and absolute log_e_ fold changes ranged from 0 to 0.2. **E)** For each chromosome, the number of male- and female-biased genes was computed, only the Y and X chromosomes were enriched for male- and female-biased genes, respectively.

Sex effects on gene expression were determined by comparing men (*N* = 1,814) and premenopausal women who did not use hormonal contraceptives (*N* = 1,594). When considering 45,418 autosomal transcripts targeting 18,495 genes, 993 transcripts from 582 genes (3.1% of all autosomal genes measured) were significantly influenced by sex (*p* < 1.2e-6, Bonferroni corrected at *p* < 0.05, FDR < 6e-5). The percentage of sex-biased genes increased when only genes with a mean expression above a certain threshold were considered. For example, a mean expression threshold of 5 (log_2_(intensity)) resulted in 5.5% sex-biased genes, and using a threshold of 9 resulted in 13.7% sex-biased genes (Figure 1A). However, there were several transcripts with low mean expression level but with a high fold change between the sexes (Figure 1B). In order to provide a comprehensive overview, we included all transcripts in the following analyses.

### Female-biased versus male-biased genes

From the sex-biased transcripts on the autosomes, 572 (57.7%) were upregulated in females (female-biased genes, Figure 1C), and 421 in males (male-biased genes, Figure 1B). For each sex-biased transcript the log_e_ fold change was computed (Figure 1D). For female-biased transcripts the fold change was computed as the mean expression in females/mean expression in males, for male-biased genes we used -mean expression in males/mean expression in females. Most absolute log_e_ fold changes were smaller than 0.08 (99%), for 22 transcripts the absolute log_e_ fold change was larger than 0.08 (6 female-biased, targeting the genes *ADM, CREB5, CNTNAP3*, *C9orf84*, *SORCS2* and *GPR109A* and 14 in men (*KANK2, CTSG, MPO, BPI, GPER, DEFA4, EPB49, C19orf62, ERG, LCN2, CEACAM8, LTF, FECH* and *LTBP1),* see Additional file 1 for sex-biased genes and corresponding fold changes and *p*-values).

On the X chromosome, 1643 transcripts from 739 genes were measured. Out of these, 127 transcripts from 51 genes were sex-biased; 103 (from 38 genes) were female-biased, and 24 (from 13 genes) male-biased. Seventeen of the corresponding log_e_ fold changes were larger than 0.08 (targeting the genes *EIF1AX, PRKX, KDM5C, ZFX, KDM6A, XIST, VSIG4, TSIX* and *SCARNA9L).* Only the log_e_ fold changes of the genes *XIST* and *TSIX* were larger than 0.5. Of the 63 transcripts targeting 26 genes on the Y chromosome, 48 transcripts from 16 genes had expression levels in men that were higher than the noise measured in women; 12 transcripts had a log_e_ fold change larger than 0.5, targeting the genes *EIF1AY, DDX3Y KDM5D, CYorf15B, CYorf15A* and *UTY.*

### Genomic location of sex-biased genes

For each chromosome we tested whether the genes on that chromosome enriched the sex-, male- or female-biased genes. At the autosomes, the percentage of sex-biased genes differed only slightly between chromosomes, ranging from 1.6% on chromosome 20 to 4.2% on chromosome 14 (Figure 1E); none of the autosomes enriched the sex-biased genes (*p* > 0.05, Fisher's exact test). The distribution of the male- and female-biased genes over the autosomes was more variable, ranging from 0.4% at chromosome 20 to 2.3% at chromosome 18 (female-biased genes), and from 0.7% at chromosome 4 to 2.4% at chromosome 22 (male-biased gene), however none of the autosomes enriched female or male-biased genes. As expected, female-biased genes were enriched for genes at the X-chromosome (5.1%), and male-biased genes for genes at the Y chromosome (61%).

### Gene ontology (GO) analysis of sex-biased genes

Female-biased genes were enriched for 52 biological process GO categories (BPGO) (*p* < 0.01, Bonferroni correction, see Additional file 2 for significant GO categories and female-biased genes therein), with as top hit immune system process (31.6% of female-biased genes are in this category, *p* < 1e-25). Significant subcategories included response to cytokine stimulus (11.3%, *p* < 1e-10), response to type 1 interferon (3.6%, *p* < 1e-7) and lymphocyte differentiation (5.6%, *p* < 1e-5)*.* Male-biased genes were not enriched for any BPGO category. The female-biased genes were enriched for 7 cellular component GO (CCGO) categories (top hits are cell surface (8.3%, *p* < 1e-6) and integral to membrane (31.3%, *p* < 1e-5)). Male-biased genes were enriched for 11 CCGO categories, with as top hit cytoplasm (73.4%, *p* < 1e-10) and significant subcategory lysome (6.4%, *p* < 1e-4). In Additional file 3 the hierarchical network structure of the significant GO categories is visualized.

### Ingenuity pathway analysis (IPA) of sex-biased genes

From the IPA biological functions, autoimmune disease (22%, FDR < 1e-11 (Fisher's exact test)) and rheumatoid arthritis (16%, FDR < 1e-11 (Fisher's exact test)) enriched the female-biased genes most significantly, among 21 other autoimmune diseases (Additional file 4). The upstream regulator lipopolysaccharide (LPS, 26%, *p* < 1e-27 (uncorrected *p*-value Fisher's exact test)) was most strongly associated with the female-biased genes, among many other regulators (Additional file 5) such as estradiol (18%, *p* < 1e-9 (uncorrected *p*-value Fisher's exact test)). Male-biased genes were most significantly enriched for genes linked to renal cancer (9%, FDR < 1e-5 (Fisher's exact test), Additional file 6). There were only few male-biased genes influenced by the same upstream regulators (Additional file 5, top hits were GATA (3%, *p* < 1e-5 (Fisher's exact test)) and HIPK2 (2%, *p* < 1e-4 (Fisher's exact test))).

### eQTL analysis of sex-biased gene expression

eQTL analysis was performed using two sample subsets of 1523 men and 1373 premenopausal women who did not take hormonal contraceptives, for which genome-wide SNP and gene expression data were available (see Methods). For each of the 993 autosomal sex-biased transcripts, eQTLs were computed for men and women separately. At a FDR of 0.01 there were 7978 cis eQTLs (*p* < 6e-05) and 514 trans eQTLs (*p* < 2e-09)) for men, and 6731 cis eQTLs (*p* < 5.2e-05) and 197 trans eQTLs (*p* < 1.8e-09)) for women. For the pooled eQTLs (9659 cis, 545 trans eQTLs) genotype-sex interactions were assessed using a mixed model that included data from men and women. At a FDR of 0.05 no significant genotype-sex interactions were observed.

### Sex-biased genes highly enrich modules of correlated transcripts

**Figure 2:**
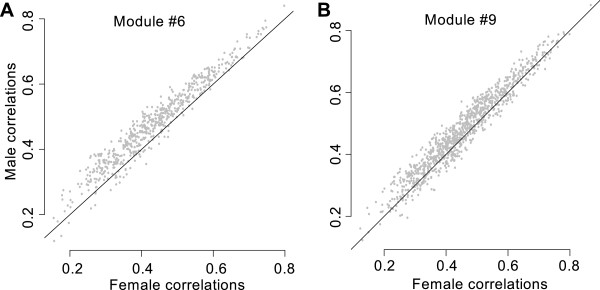
**Between transcript correlations are higher in males than in females for 2 modules.** WGCNA (Weighted Gene Co-Expression Network Analysis) resulted in 9 modules with correlated transcripts, two of which were highly enriched for female-biased genes, and 3 for male-biased genes. From the latter three, two modules contained genes from which the pair-wise correlations were stronger in males compared to females. **A)** Module #6 contained 45 genes, 76% of the correlations computed in males (y axis) were larger than those computed in females (x axis). **B)** Module #9 contained 35 genes, 92% of the correlations computed in males (y axis) were larger than those computed in females (x axis).

Weighted Gene Co-Expression Network Analysis (WGCNA) [28] was used to identify modules of correlated transcripts, for men and women separately. Both analyses resulted in 9 modules with >70% transcript overlap between the male and the corresponding female module for 8 of the 9 modules (Additional file 7). One module had only ~40% overlap. Thus, gene expression correlation structure is similar between men and women, and here we focus on properties of the intersection of the overlapping modules. Interestingly, 7 of these intersected modules were highly enriched with female-biased or male-biased genes. There were three modules with more than 30% male-biased genes, and two modules with more than 30% female-biased genes. The modules were highly enriched for several GO terms (Additional file 7). We calculated the pairwise transcript correlations within each intersected module or men and women separately. For two modules containing male-biased genes the correlations were significantly stronger in males than in females (76% of the correlations were stronger in module #6, and 92% in module #9, Figure 2A & B respectively). Thus, these modules contained around 30% of male-biased genes, but also the majority (> 75%) of the interactions in the module were stronger in males compared to females.

### Evolution rates of sex-biased genes

To test whether sex-biased genes have evolved faster than non sex-biased genes, we tested for enrichment in two sets of genes that were previously identified as rapidly evolving: 244 genes from the Human PAML Browser [29] and 40 genes from a study comparing human and chimpanzee genomes [30]. Sex-biased, male-biased and female-biased genes were not enriched for any of the two gene sets (Fisher's exact test, *p* > 0.05). Next, we tested whether dN, dS and dN/dS (the evolution rates) as provided by [31] were different in sex-biased, male-biased and female-biased genes compared to non sex-biased genes, but found no significant differences (all *p* > 0.05, Wilcoxon rank test).

### Tissue specificity of sex-biased genes

We downloaded analysis results of two human studies that identified sex-biased genes in muscle [22] and in liver [21]. In muscle, 63 sex-biased genes were identified on the autosomes which were enriched with the sex-biased genes we identified (8 genes identified in both tissues, *p* < 0.01 (Fisher's exact test), Additional file 8). On the X chromosome 5 genes were identified as sex-biased in muscle, of which 4 were also identified in blood (*p* < 0.001 (Fisher's exact test), Additional file 8). In liver, 862 sex-biased genes were identified on the autosomes which were enriched with the sex-biased genes we identified (36 genes identified in both tissues, *p* < 0.05 (Fisher's exact test), Additional file 8). On the X chromosome 50 genes were identified as sex-biased in liver, of which 18 were also identified in blood (*p* < 1e-9 (Fisher's exact test), Additional file 8).

### Sex-biased genes in postmenopausal and hormonal contraceptive using women

**Figure 3:**
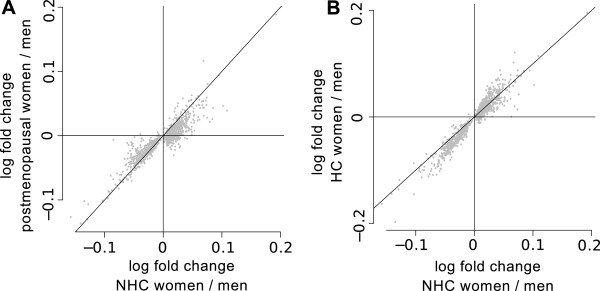
**Sex-differences in gene expression are increased by the use of hormonal contraceptives, and decreased during menopause.** Women were divided in three groups: postmenopausal, hormonal contraceptive using (HC), and non hormonal contraceptive using (NHC) women. For the 993 sex-biased transcripts identified in the comparison between males and NHC women, fold changes were computed for the difference between the three groups of women and the men. Positive fold changes are from female-biased genes, negative fold changes correspond to male-biased genes. **A) ** Fold changes are for 80% larger in NHC women as compared to postmenopausal women. **B)** Fold changes in HC women are for 66% larger than those observed in NHC women, and the negative fold changes (male-biased genes) were for 88% larger in HC women.

To examine whether sex differences in gene expression depend on hormonal status, sex effects were computed by comparing men (*N* = 1,814) with postmenopausal women (*N* = 740) and women using hormonal contraceptives (HC women, *N* = 1,093). On the autosomes, there were 697 transcripts differentially expressed between postmenopausal women and men. From these 697 transcripts (369 female-biased and 328 male-biased) 236 overlapped with the 993 sex-biased transcripts identified in non-hormonal contraceptives using premenopausal (NHC) women. When comparing the HC women with men, a much larger number of 2,125 differentially expressed transcripts were identified (1,157 female-biased, 968 in male-biased). From these transcripts, 755 were overlapping with the 993 sex-biased transcripts identified in NHC women. For the 933 transcripts identified in NHC women, log_e_ fold changes were computed for the difference between each of the three groups of women (NHC, HC, postmenopausal) compared to men. When comparing these fold changes between postmenopausal and NHC women, it became clear that most of the fold changes have the same sign (85% in total, 99% of the negative fold changes) but that the fold changes in NHC women are larger than those in postmenopausal women for 80% of the transcripts (Figure 3A). Also the fold changes of NHC women and HC women often have the same sign (96%), and the fold changes of HC women were often larger than those observed in NHC women (66% of all fold changes, 88% of the negative fold changes, Figure 3B). This shows that many gene expression differences between women and men become smaller when women reach menopause, and are larger when women use hormonal contraceptives, which reinforces the role of estrogen in regulating sex-biased genes.

### Age specific sex effects on gene expression

Age has a strong influence on gene expression [32]. To examine whether sex effects on gene expression are age-range specific, we separately analyzed the data for three age groups (men versus premenopausal women who did not use hormonal contraceptives, age ranges 17-30 (*N* = 1047), 31-40 (*N* = 1191) and 41-88 (*N* = 1170)). In these 3 age groups we identified 49, 103 and 34 autosomal sex-biased genes respectively (*p* < 1.2e-6, Additional file 9), which overlapped for >98% with the sex-biased genes identified in the total sample (with same direction of effect). The three sets of sex-biased genes identified in these age groups overlapped to a lesser extent with each other (>38%, Additional file 9). However, the fold changes between men and women of the sex-biased genes identified in the total sample were highly concordant between age ranges (Additional file 10), suggesting that the identified sex effects occur at all ages, but that some effects may be stronger at a certain age or may not have been identified due to reduced power in the smaller groups of selected ages.

## Discussion

At the DNA autosomal sequence level sexes do not differ, as established by a well-powered meta-analysis [33], suggesting an important role for higher molecular levels, such as the transcriptome, in the manifestation of sexual dimorphisms. Indeed, animal studies have shown that the transcriptome is highly differential between sexes [14,15,34,35]. In humans, gene expression differences have been reported in liver [21], lymphoblastoid cell lines [19,20], and muscle [22], but only in studies with relatively small sample sizes (*N* < 250). Here we analyzed the sex differences in the peripheral blood transcriptome by assessing 47,122 probe sets targeting 19,250 genes genome wide in a well-characterized large Dutch cohort (*N* = 5,241) taking into account the impact of hormonal contraceptive use and menopausal status in women.

### Number of female- and male-biased genes

On the autosomes, we identified 582 genes (3.1% of all genes measured) that were differentially expressed between men and premenopausal women not using hormonal contraceptives. Of these genes, 57.7% were female-biased. The autosomes had rather similar proportions of sex-biased genes indicating the sex-biased genes can be found equally frequent across the entire genome, as opposed to what was found in liver [21] where several chromosomes enrich sex-biased genes. It is important to note that the filter criteria used for selecting probe sets highly influences the number of sex-biased genes; the percentage of sex-biased genes increased with the threshold for mean expression level from 3.1% up to 13.7%. Importantly, we have shown that hormonal contraceptives and menopause status, which were not taken into account in previous studies in humans, highly influence the number and effect sizes of sex differences in gene expression. Although it has been indicated that the percentage of sex-biased genes in non-human vertebrates is highly tissue dependent (e.g. ranging from 13.6% in the brain to 72% in the liver [14,15]), our described range of 3.1-13.7% for sex-biased genes is comparable to that found in human liver (3.7%, [21]). Peripheral blood consists of a mixture of blood cell types (the main types are lymphocytes, neutrophiles and monocytes), hence the sex differences we identified must either be present in all subcell types or, when present in only one cell type, strong enough to be observed in the accumulative measurement. By stratifying the sample into three age groups we showed that the size of the sex effects may be age dependent for some genes, but the direction of the effects are highly concordant between age groups.

We found a significant but small overlap of sex-biased autosomal genes identified in peripheral blood with those previously identified in muscle or liver, further confirming substantial tissue specificity of sex-biased genes. Across tissue circulating exosomes contain RNA and could contribute to the overlapping expression profiles between muscle, liver and blood [36]. Sex-biased X chromosome genes showed must larger overlap between tissues, indicating that escape from X-inactivation is highly similar between tissues. Previous studies have reported that sex-biased genes may evolve more rapidly than average in vertebrates [12], human brain [37] and liver [21]. However, sex-biased genes in the peripheral blood transcriptome identified in our study did not include enrichment of fast evolving genes. In women, most genes on one X chromosome are not expressed due to X chromosome inactivation [38]. Some genes escape X-inactivation and are expressed from both X chromosomes [7]. We showed that in peripheral blood the X chromosome is enriched for female-biased genes; 5.1% of the genes measured on the X chromosome are female-biased. This percentage, however, is only slightly higher than the average percentage identified at the autosomes (3.1%), which shows a major role of autosomal genes in sex-specific gene expression.

### The role of estradiol in gene expression sex differences

Estrogen is the primary female sex hormone and estrogenic activity is present at about two fold increased concentration in women as compared to men*.* Estradiol, the predominant estrogen in terms of absolute serum levels, activates estrogen receptors that bind to DNA sequences to activate or suppress gene expression, and many efforts have been made to find its target genes (up to 5000) in MCF-7 cancer cell line [39-41] because of its role in breast cancer [42]. Here we show that in peripheral blood 18% of the identified sex-biased genes are known to be regulated by estradiol, and several additional findings suggest that the sex difference in estrogen levels underlie multiple sex differences in gene expression. First, from the 20 genes with high male/female fold changes, 7 are involved in common diseases and influenced by estrogen; *GPER* (g protein-coupled estrogen receptor-1, related to cancer [43]), *ADM* (coding for the peptide adrenomedulin, the main vasodilatory peptide involved in cardiovascular disease [44-46], *LTF* (lactoferrin, essential for the innate immune system and involved in cancer [47,48]), *LCN2* (lipocalin-2, innate immune system and cancer related [49]), *MPO* (myeloperoxidase), a biomarker for cardiovascular disease risk [50], *ERG* (Ets Related Gene, proposed as a mediator of estrogen effect on prostate cancer [51], *LTBP1* (latent-transforming growth factor beta-binding protein 1, linked to coronary heart disease [52]. This suggests that these genes mediate the effect of estrogen and thereby may contribute to the sex differences in the related diseases. Second, we showed that the sex differences in gene expression depend largely on the hormonal status of the subgroup of women considered. In postmenopausal women, in which estradiol levels are similar to those in men, we identified fewer sex-biased genes with smaller effect sizes as compared to premenopausal women. In hormonal contraceptive using women, with increased estradiol levels, we identified more sex-biased genes and larger effect sizes as compared to women not using hormonal contraceptives. Interestingly, the change in effect size was present for more than 65% of the female-biased genes, and for more than 85% of the male-biased genes. This gives an indication of the amount of sex-biased genes affected by estradiol, which is much higher than currently known from literature (IPA, 15% of sex-biased genes are known to be regulated by estradiol). In liver, sex differences in gene expression are mainly caused by sex-specific growth hormone secretion [21,53]. Growth hormones are regulated by estrogen [54,55], hence the effect of estrogen on sex-specific gene expression in peripheral blood may also be mediated by growth hormone secretion.

### Immune system processes predominant in female-biased genes

The immune system function is known to be different between sexes; women produce more vigorous immune reactions and are more prone to autoimmune diseases [56]. Here we identified a large number of genes that potentially contribute to the immune system sex differences; 31.6% of female-biased genes are in the GO category immune system process. From the 95 female-biased genes linked to the immune system, 45 are regulated by estradiol, which confirms the role of estrogen in the sex-specific immune system functioning [57]. Most interestingly, Ingenuity Pathway Analysis revealed that female-biased genes are highly enriched for genes involved in the toll-like receptor (TLR4 and TLR3 pathways, known as LPS and poly I:C response patterns) driven innate immune defense, suggesting some intrinsic innate immune activity sex differences. Increased female expression of immunoglobulin is reflective of concomitant more active humoral immune activity. These functions are compatible with an activated leukocyte, cytokine production and type 1 interferon activity observed in the GO enrichment analysis and might explain why women are more resistant to certain infections, and suffer a high incidence of autoimmune diseases compared to men [2]. For example, rheumatoid arthritis occurs almost twice as often in women as in men [58]. Female-biased genes were enriched for genes linked to rheumatoid arthritis, including the gene *IL6R,* which is a well-known target in rheumatoid arthritis treatment [59]. The identified female-biased genes provide a framework for future research to unravel the mechanism of sex-biased immune regulation and autoimmune diseases.

### Annotation of male-biased genes

Surprisingly, male-biased genes were not enriched for GO categories, and thus serve a wide variety of biological functions. In IPA, however, male-biased genes were most significantly enriched for genes linked to renal cancer, including the well established renal cancer gene CSF1R [60]. It is notable that a recent meta-analysis on sex differences in renal cell cancer presentation and survival showed a ratio of 1.65 of renal cell carcinoma for males compared to females [61]. The cellular component GO categories indicate the part of a cell at which a gene product is located. Topographical categorization revealed that male-biased gene products occur more often intracellularly, in particular at the cytoplasm, whereas female-biased genes occur more often integral to the membrane.

### Sex-specific eQTLs do not underly sex-biased gene expression

A previous study (in a smaller sample than the current one) showed that a substantial amount of eQTLs is sex-specific, but not for eQTLs from genes with sex-biased expression [26]. Here we confirm this finding by showing that for the sex-biased genes there were no significant eQTL-sex interactions. This shows the importance of other factors, such as estradiol and other hormones, in causing gene expression sex differences.

### Sex-biased genes in modules of correlated transcripts

WGCNA analyses resulted in highly similar modules of correlated transcripts for men and women, similar to findings in mice [18]. The 9 modules were highly enriched for male or female-biased genes, indicating that sex-biased genes play an important role in the major gene expression networks. Module #2 and #3 contained each more than 30% female-biased genes and were enriched for the GO category immune system response, which shows that immune system genes operate in correlated groups that are partially sex-biased. Module #9 contained 31.4% male biased genes, enriched the GO category immune response (37%) and contained 92% stronger pairwise correlations in men than in women. This module contained the interleukin receptor *IL2B* gene, and IPA analysis showed that 11 of the 35 genes in this module are known to be regulated by the cytokine IL2, and 16 of them are related to cancer (Additional file 11) including the female-biased genes *PRF1* and *GZMH* essential for natural killer (NK)-cell cytotoxicity [62,63]. Module #6 contained 37.8% male-biased genes, was enriched for the GO term coagulation (50%) and 76% of the pairwise correlations in this module are higher in men than in women. IPA analysis shows that from this module 16 genes are regulated by TGFB1 (Additional file 11), and 17 genes are related to heart or vascular disease, including the male-biased genes *PTGS1* (coding for COX-1, which is inhibited by aspirin [64] that has a protective effect on cardiac events [65]), *ITGA2B*, *ITGB3*, *F13A* and *GP1BA* which are candidate stroke risk genes [66]. This suggests that the modules #9 and #6 may play a role in the sex differences in cancer and cardiovascular disease, respectively.

## Conclusions

We showed that sex-biased genes occur in large numbers throughout the human peripheral blood transcriptome, suggesting an important role of sex-specific gene expression in sexual dimorphisms. Estrogen appears to be a key regulator of sex-biased genes, also shown by the effect of menopause and hormonal contraceptives on gene expression sex differences. Sex-biased genes are highly enriched with genes linked to common diseases and may contribute to sex-differences in these diseases. Understanding the molecular mechanisms behind sex inequalities can lead to new insights into sex-specific pathophysiology and treatment opportunities.

## Methods

### Subjects

The two parent projects that supplied data for this study are large-scale longitudinal studies: the Netherlands Study of Depression and Anxiety (NESDA) [67] and the Netherlands Twin Registry [68]. NESDA and NTR studies were approved by the Central Ethics Committee on Research Involving Human Subjects of the VU University Medical Center, Amsterdam (IRB number IRB-2991 under Federalwide Assurance 3703; IRB/institute codes, NESDA 03-183; NTR 03-180), and all subjects provided written informed consent. The sample consisted of 5391 subjects (before QC), 3327 participants from NTR (2 MZ triplets, 708 MZ twin pairs, 658 DZ twin pairs, 338 siblings from these twins and 251 unrelated individuals) and 2064 unrelated participants from NESDA. The age of the participants ranged from 17 to 88 years (mean 38, SD 13) and 65% of the sample was female. As part of the NESDA and NTR biobank protocols, data on menopause status and medication use, including hormonal contraceptives were collected in all participants.

### Blood sampling, RNA and DNA extraction

The NTR and NESDA blood sampling and RNA extraction procedures have been described in detail previously [69,70]. In short; for NTR, venous blood samples were drawn between 0700-1100 after an overnight fast and usually in the subjects’ homes. Within 20 minutes of sampling, heparinized whole blood was transferred into PAXgene Blood RNA tubes (Qiagen) and stored at -20°C. The PAXgene tubes were shipped to the Rutgers University Cell and DNA Repository (RUCDR), USA. Average time between blood sampling and RNA extraction was 211 weeks (included in mixed model for gene expression). Upon registration of samples, RNA was extracted using Qiagen Universal liquid handling system (PAXgene extraction kits as per the manufacturer's protocol).

From the NESDA subjects, serial venous whole blood samples were obtained (8–10 am, after overnight fasting) in one 7-mL heparin-coated tube (Greiner Bio-One, Monroe, North Carolina). Between 10 and 60 min after blood draw, 2.5 mL of blood was transferred into a PAXgene tube (Qiagen, Valencia, California). This tube was kept at room temperature for a minimum of 2 hours and then stored at -20°C. Average time between blood sampling and RNA extraction was 113 weeks (included in mixed model for gene expression). Total RNA was extracted at the VU University Medical Center (Amsterdam) according to the manufacturer’s protocol (Qiagen) as described previously [70].

For both NESDA and NTR samples high molecular weight genomic DNA was isolated from frozen blood in EDTA tubes using Puregene DNA isolation kits (Qiagen).

### Gene expression measurements

Gene expression assays were conducted at the Rutgers University Cell and DNA Repository (RUCDR, http://www.rucdr.org). RNA quality and quantity was assessed by Caliper AMS90 with HT DNA5K/RNA LabChips. RNA samples that showed abnormal ribosomal subunits in the electropherograms were removed. NTR and NESDA samples were randomly assigned to plates with seven plates containing subjects from both studies to better inform array QC and study comparability. For cDNA synthesis, 50 ng of RNA was reverse-transcribed and amplified in a plate format on a Biomek FX liquid handling robot (Beckman Coulter) using Ovation Pico WTA reagents per the manufacturer’s protocol (NuGEN). Products purified from single primer isothermal amplification (SPIA) were then fragmented and labeled with biotin using Encore Biotin Module (NuGEN). Prior to hybridization, the labeled cDNA was analyzed using electrophoresis to verify the appropriate size distribution (Caliper AMS90 with a HT DNA 5 K/RNA LabChip). Samples were hybridized to Affymetrix U219 array plates (GeneTitan) to enable high-throughput gene expression profiling of 96 samples at a time. The U219 array contains 530,467 probes for 49,293 transcripts. All probes are 25 bases in length and designed to be “perfect match” complements to a designated transcript. Array hybridization, washing, staining, and scanning were carried out in an Affymetrix GeneTitan System per the manufacturer’s protocol.

### Genome-wide SNP measurements and QC

Genotyping was conducted using Affymetrix Genome-Wide Human SNP Array 6.0 containing 931,946 SNPs, per the manufacturer’s protocol. The resulting data were required to pass standard Affymetrix QC metrics (contrast QC > 0.4) before further analysis. SNP QC included removal of SNPs for non-unique mapping of probe sequences to NCBI Build 37/UCSC hg19, low minor allele frequency (< 0.005), substantial deviation from HapMap3 CEU founder allele frequencies, deviation from Hardy-Weinberg equilibrium (p_HWE_ < 1×10^-8^), and high missingness (> 0.05). After genotyping QC, 666 K autosomal SNPs were available. Subjects were eliminated from analysis for high missingness (> 0.05), outlying genome-wide homozygosity or ancestry, discrepant genetic and phenotypic sex, or twin relatedness not consistent with monozygosity or dizygosity.

### Gene expression QC

Gene expression data were required to pass standard Affymetrix QC metrics (Affymetrix expression console) before further analysis. Probes were removed when their location was uncertain or if their location intersected a polymorphic SNP (dropped if the probe oligonucleotide sequence did not map uniquely to hg19 or if the probe contained a polymorphic SNP based on HapMap3 and 1000 Genomes project data). Expression values were obtained using RMA normalization implemented in Affymetrix Power Tools (APT, v 1.12.0). First, 70 samples with array results inconsistent with the phenotypic database were removed (inconsistent sex based on chr X and chr Y probe sets). Second, we used the pairwise correlation matrix of expression profiles across all arrays for additional QC. These quantities were expressed in terms of median absolute deviations to provide a sense of scale. We used:

$$D_{i}=\frac{\left|r_{i}‒r\right|}{\text{median}\;{\left(\left|r_{k}‒r\right|\right)}_{k=1...N}}$$

With *r*_*i*_ the average of correlations for sample *i,* and *r* the average of all correlations*.* Larger values of *D* corresponded to poor quality; 80 samples with *D* > 5 were removed, decreasing the final number of subjects to 5,241.

### Mixed models for gene expression

Linear mixed models allow for the correction for the presence of twin families in a sample [71]. For each of the 47,122 probe sets a mixed model was fit with gene expression as dependent variable. Independent model covariates were selected based on significance of the variable in the fitted mixed models. Several covariates that did not come out significantly were not included in the final model (alcohol use, education level, time between RNA amplification and RNA fragmentation, time between RNA fragmentation and RNA hybridization). Inclusion of depression status and psychotropic medication use as covariates in the mixed model did not affect the principle findings. Fixed effect covariates included in the final model were sex, age, body mass index (BMI, weight/height 2 in kg/m), smoking status (yes/no current smoking), D (see above), hemoglobin (mmol/L), group (NTR or NESDA), time of blood sampling, month of blood sampling, time between blood sampling and RNA extraction, and the time between RNA extraction and RNA amplification. Random effects were plate, well, family ID and zygosity (one factor for each monozygotic twin pair, for each other individual different factors [71]). In Additional file 12, for each of the variables the amount of probe sets for which the variable was significant is denoted. Mixed models and resulting p-values were computed using the R function lmer from the package lme4.

### eQTL analysis

eQTL analysis was first performed in a screening step by MatrixeQTL [72]. Prior to eQTL analysis for each gene expression probeset the data was transformed into a normal distribution using an inverse quantile normal transformation. Genotypes were coded as 0, 1 or 2 and for each SNP-transcript pair a linear regression model was fitted including the covariates sex, age, body mass index, smoking status, D (see above), hemoglobin (mmol/L), group (NTR or NESDA), time of blood sampling, month of blood sampling, time between blood sampling and RNA extraction, time between RNA extraction and RNA amplification, plate and well plus three principle components (PCs) from the genotype data [73] and 5 PCs from the transformed expression data. Cis-eQTLs are transcript-associated SNPs with distance < 1 Mb of transcript site. The trans-eQTLs are the complementary set of SNPs. In the screening step, males and woman were screened using MatrixeQTL as if the individuals were all unrelated. Benjamini-Hochberg q-value estimation was performed separately for cis- and trans-eQTLs. For each of the 993 autosomal sex-biased transcripts eQTLs were selected for men and women separately, and then pooled. For these eQTLs genotype-sex interactions were assessed using the full mixed model that included both men and women, with as independent variables genotype, sex, their interaction and the other covariate also used in the mixed model for gene expression (see above).

### GO category enrichment

To test whether Gene Ontology [74] categories enriched sex-biased genes we used hypergeometric tests implemented in BINGO software [75]. The reference gene set consisted of all genes measured by the U219 microarrays.

### WGCNA

The correlation structure of gene expression was examined using unsigned co-expression networks constructed using the WGCNA package in R [28]. Of all 47,122 probes a single probe of highest mean expression per gene was selected to be included in the network analysis using the CollapseRows function in WGCNA, resulting in the inclusion of 19,249 genes in the network. The choice of the probe of highest mean expression per gene has been shown to yield robust analysis across data sets [76]. The network construction for each entire data set was performed in a single block of maximum size 20,000 genes using the blockwiseModules function in WGCNA [28]. Using this block size in WGCNA ensured the theoretical advantage that the genes did not have to be pre-clustered by WGCNA. The network adjacency matrix is the gene pair-wise correlation matrix raised to the power of 6, chosen based on the scale-free topology criteria [77]. Rather than just using adjacency weights between genes, the topological overlap measure (TOM) is computed from the adjacency matrix. For each pair of genes, TOM is the adjacency weights of all the paths between the genes of length at most two (i.e. the genes are directly connected or have one gene between them) scaled by the minimum connectivity of the either gene. The topological overlap dissimilarity, defined as 1-TOM, is used for the average linkage hierarchical clustering algorithm. The resultant clustering tree is used to define the modules from its branches using the hybrid dynamic tree cutting algorithm [28]. The minimum module size was set to 30 and the cut-off for merging modules was set to 0.25. Each module is then characterized by its eigengene, the first principal component of the module expression data, which accounts for the greatest variation of the expression levels in the module. Genes were removed from modules if the correlations between their expression values and the module eigengenes were too low (less than 0.3). Modules were merged if the correlation between their eigengenes was high.

### Availability of supporting data

Gene expression data used for this study will be available at dbGaP, accession number phs000486.v1.p1 (http://www.ncbi.nlm.nih.gov/projects/gap/cgi-bin/study.cgi?study_id=phs000486.v1.p1).

## Abbreviations

GO: Gene ontology; BPGO: Biological process GO; CCGO: Cellular component GO; WGCNA: Weighted gene co-expression network analysis; HC women: Women using hormonal contraceptives; NHC women: Non-hormonal contraceptives using women; IPA: Ingenuity pathway analysis.

## Competing interests

The authors declare that they have no competing interests.

## Author’s contributions

RJ and SB performed analysis. AB and JT generated molecular data. GW, VM and GvG provided data management support, JJH, YM and HM provided genotype data QC. RJ, WP JV, CLV, EG, JS, FW, PS, DB and BP wrote the manuscript. All authors read and approved the final manuscript.

## Supplementary Material

Additional file 1 **Sex-biased genes and corresponding p-values and log**_**e **_**fold change.** Click here for file

Additional file 2 Significant GO categories for female-biased genes. Click here for file

Additional file 3 **Hierarchical structure of Gene Ontology categories enriching male or female-biased genes.** GO enrichment analysis for female (A, B) and male-biased genes (C) in the main categories biological process (A, C) and cellular component (B). GO categories are represented as circles, size of circle shows total amount of genes in this category, color of circle codes for p-value for enrichment (the color white means not significant). An arrow pointing from category A to category B means that B is a subcategory of A. GO categories containing few genes with no further subcategories often occur at the outside of the network, and provide the most specific classification, such as categories response to type 1 interferon, lymphocyte differentiation, anti-apoptosis and lysome. Click here for file

Additional file 4 Autoimmune diseases enriching female-biased genes. Click here for file

Additional file 5 Upstream regulators associated with sex-biased genes. Click here for file

Additional file 6 Male biased genes associated with renal cancer. Click here for file

Additional file 7 Characterization of modules. Click here for file

Additional file 8 Sex-biased genes identified in multiple tissues. Click here for file

Additional file 9 Sex-biased genes in three age ranges. Click here for file

Additional file 10 **Fold changes of sex-biased genes in three age groups.** Subject were divided in three age groups: 18-30, 31-40, and 41-88. For the 993 sex-biased transcripts identified in the full sample, fold changes between men and women were computed within the three groups and plotted against each other. The figures show that the fold changes are highly concordant between age ranges. Click here for file

Additional file 11 Characterization of genes in Modules #6 and #9. Click here for file

Additional file 12 Covariates selected for mixed model. Click here for file
