# Supplementary figures and images for: Sex differences in the human peripheral blood transcriptome
Source: BMC Genomics. 2014 Jan 17;15:33. doi: 10.1186/1471-2164-15-33 (PMC3904696; doi:10.1186/1471-2164-15-33)

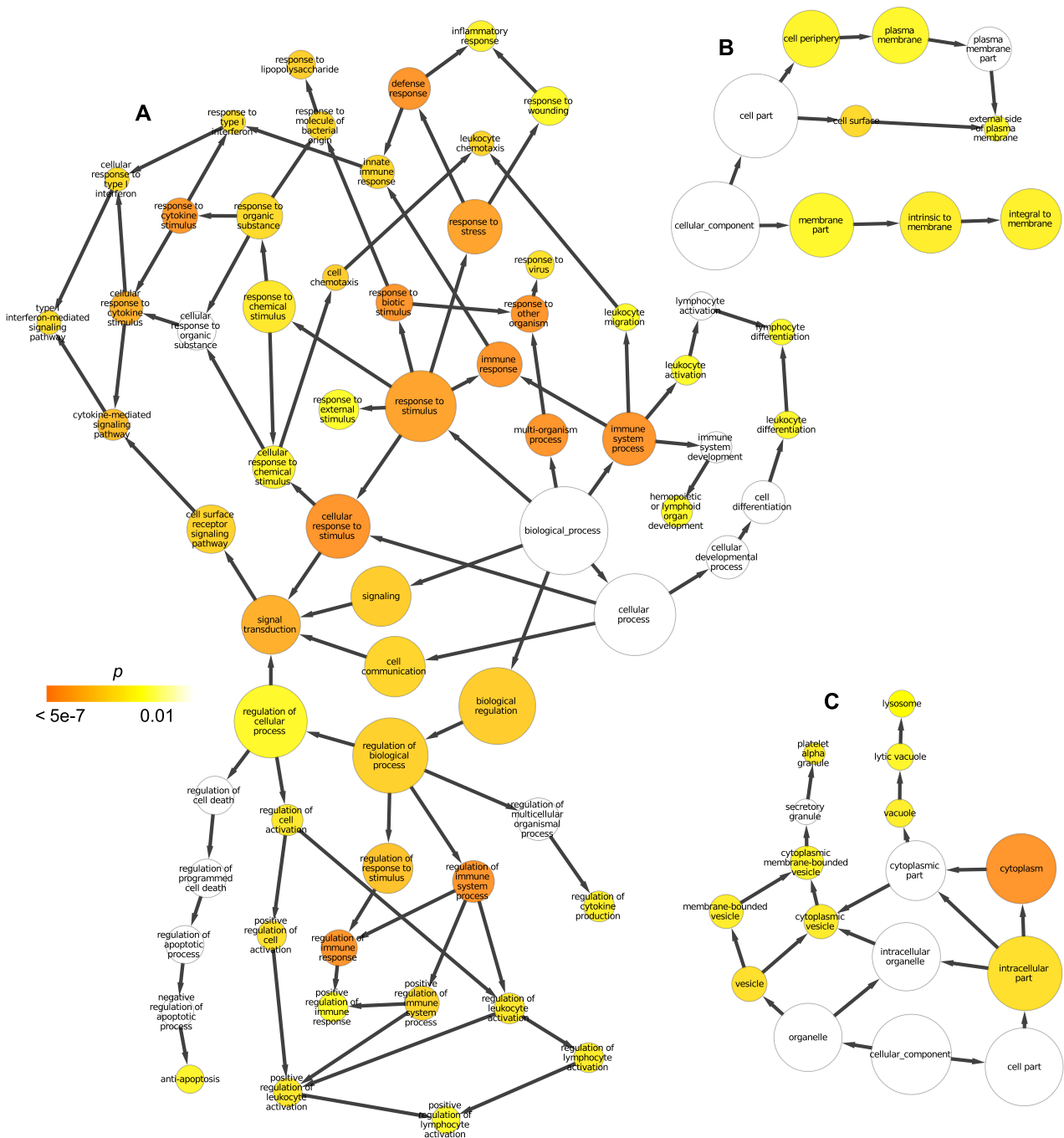

Supplement: Additional file 3 — Hierarchical structure of Gene Ontology categories enriching male or female-biased genes. GO enrichment analysis for female (A, B) and male-biased genes (C) in the main categories biological process (A, C) and cellular component (B). GO categories are represented as circles, size of circle shows total amount of genes in this category, color of circle codes for p-value for enrichment (the color white means not significant). An arrow pointing from category A to category B means that B is a subcategory of A. GO categories containing few genes with no further subcategories often occur at the outside of the network, and provide the most specific classification, such as categories response to type 1 interferon, lymphocyte differentiation, anti-apoptosis and lysome. [file 1471-2164-15-33-S3.pdf]

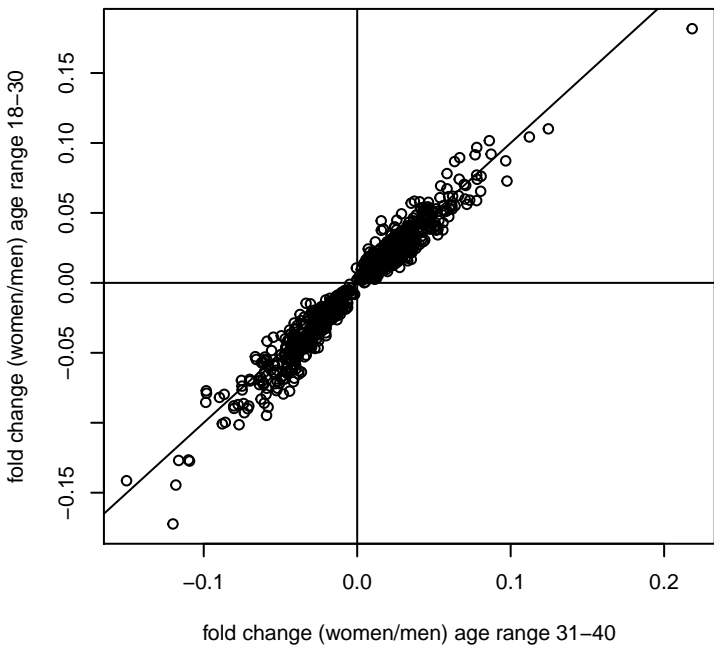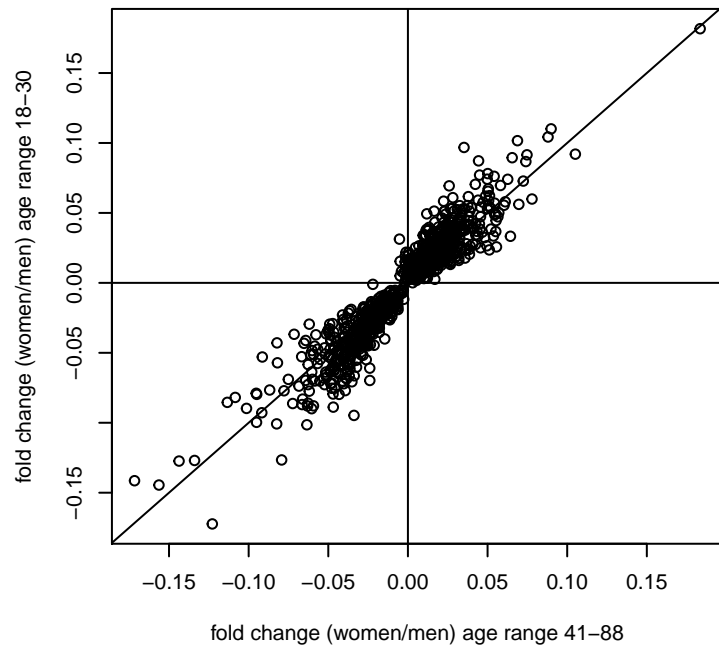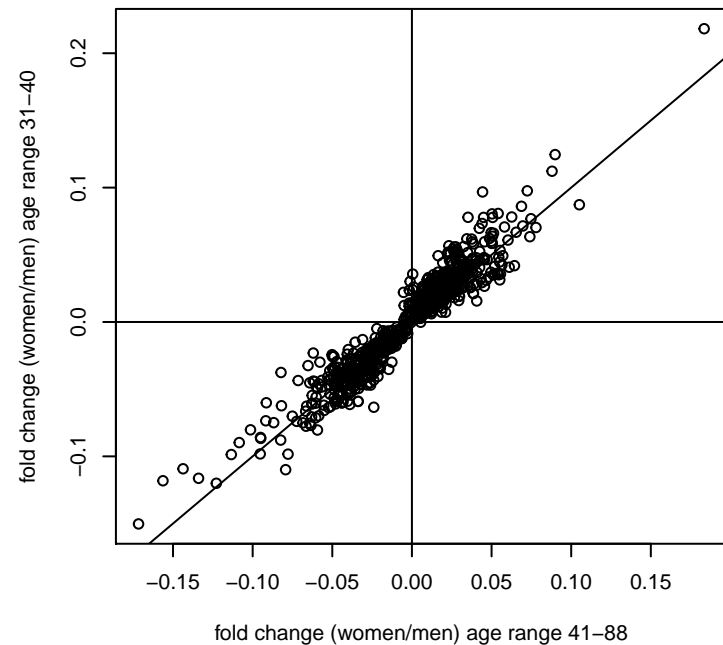

Supplement: Additional file 10 — Fold changes of sex-biased genes in three age groups. Subject were divided in three age groups: 18-30, 31-40, and 41-88. For the 993 sex-biased transcripts identified in the full sample, fold changes between men and women were computed within the three groups and plotted against each other. The figures show that the fold changes are highly concordant between age ranges. [file 1471-2164-15-33-S10.pdf]
